# Aeroallergen Der p 2 promotes motility of human non-small cell lung cancer cells via toll-like receptor-mediated up-regulation of urokinase-type plasminogen activator and integrin/focal adhesion kinase signaling

**DOI:** 10.18632/oncotarget.14514

**Published:** 2017-01-05

**Authors:** Chun-Hsiang Lin, Hui-Han Lin, Cheng-Yi Kuo, Shao-Hsuan Kao

**Affiliations:** ^1^ Institute of Biochemistry, Microbiology and Immunology, Chung Shan Medical University, Taichung, Taiwan; ^2^ Surgical Department Cardiovascular Division, China Medical University Hospital, Taichung, Taiwan; ^3^ Department and Graduate Institute of Biology and Anatomy, National Defense Medical Center, Taipei, Taiwan; ^4^ Clinical Laboratory, Chung Shan Medical University Hospital, Taichung, Taiwan

**Keywords:** Der p 2, non-small cell lung cancer, integrin, urokinase-type plasminogen activator, urokinase-type plasminogen activator receptor

## Abstract

House dust mite (HDM) allergens are one of the major causes leading to respiratory hypersensitiveness and airway remodeling. Here we hypothesized that a major HDM allergen Der p 2 could increase cell motility and invasiveness of non-small cell lung cancer (NSCLC) cells. Our results showed that low dose (1 and 3 μg/mL) recombinant Der p 2 protein (DP2) enhanced the migration and invasiveness of human NSCLC cell A549, H1299 and CL1-5, but nonsignificantly altered their growth. Further investigation revealed that integrin αV level was increased and its downstream signaling including focal adhesion kinase (FAK) and paxillin were activated in A549 cells exposed to DP2. In parallel, DP2 also activated the FAK-associated signaling effectors such as Src, phosphatidyl inositol 3-kinase (PI3K), AKT, p38 mitogen-activated protein kinase (P38), extracellular signal-regulated kinase 1/2 (ERK1/2) and c-Jun N-terminal kinase (JNK). Our findings also revealed that DP2 increased expression level of urokinase type plasminogen-activated kinase (uPA) and uPA receptor (uPAR), and subsequently enhanced the binding of uPAR to integrin αV. Moreover, the involvement of toll-like receptor 2/4 (TLR2/4)-triggered ERK1/2 activation in the increased expression of uPA and uPAR was also demonstrated. Collectively, these findings indicate that DP2 can enhance cell motility and invasiveness of NSCLC cells, attributing to TLR2/4-ERK1/2 activation, increased uPA and uPAR expression, enhanced binding of uPAR to integrin αV, and the consequent FAK signaling cascades. Thus, we suggest that DP2 may exacerbate NSCLC via promoting metastatic ability of carcinoma cell.

## INTRODUCTION

Lung cancer is the most life-threatening malignancy in Taiwan and causes more than 7,000 deaths annually [1]. Typical treatments for lung cancer include surgery, chemotherapy, radiation therapy and the combinations of the above. Good prognosis is usually expected for the patients who have received optimal treatments at the early stage. Non-small cell lung cancer (NSCLC) is the major type of lung cancer that accounts for > 75% of all lung cancer cases [2]. Most of the NSCLC patients are diagnosed at advanced stages with multiple organ metastases, the major cause leading to death, and their survival rates are lower as comparing to the other types of lung cancer [3].

Tumor metastasis is a multistep process including various cellular and molecular mechanism changes, conducting detachment of tumor cells from *in situ* carcinoma cell, migration and invasion through the basement and extracellular matrix (ECM), intravasation into the blood flow and the subsequent extravasation and growth at distant organs [4]. Diverse cellular signaling molecules, particular kinases and phosphatases, are coordinately regulated during cell migration and invasion, the indispensable initial step for metastasis. Among the signaling molecules, focal adhesion kinase (FAK), a non-receptor tyrosine kinase involved in ECM/integrin-mediated signaling pathways, is known to associate with malignant transformation, progression, and tumor metastasis [5]. Activated integrin binding to its ligand contributes to the formation of focal adhesion complex that activates FAK and Src family kinases, and subsequently initiates multiple downstream signaling pathways including Ras/mitogen-activated protein kinase kinase (MEK)/extracellular regulated protein kinase (ERK) cascades that promote cell migration and invasion [6].

The house dust mite (HDM), predominantly *Dermatophagoides pteronyssinus* and *Dermatophagoides farina*, has been considered as a major indoor risk factor for respiratory hypersensitiveness and atopic asthma [7]. Among the 24 groups of allergens that have been characterized and identified from HDM, group I and group II allergens have been reported to possess the highest IgE-binding frequency [8]. In contrast to to group I-HDM allergens that possess cysteine protease activity, group II-HDM allergens such as Der p 2 belong to non-proteolytic proteins. Although Der p 2 shares both structural homology and similar biological activity with MD-2 protein, Der p 2 can trigger innate immunity via activating toll-like receptor signaling without binding to lipopolysaccharide [9]. Der p 2 has also been reported to enhance nitric oxide (NO) production by alveolar macrophages via a non-enzymatic pathway, causing lung inflammation and epithelial injury [10]. In addition, Der p 2 can induce apoptosis of bronchial epithelial cell [11], evoke bystander activation of autoimmune B cell [12], promote epithelial-to-mesenchymal transition (EMT)-associated cell motility [13], and upregulate expression of epithelial tight junction molecules [14].

Although the association between respiratory allergy and lung cancer progression has received increased interest, the effects of aeroallergen on lung carcinoma cells are rarely explored. Integrin and its downstream signal effector focal adhesion kinase (FAK) have been widely reported involving in metastasis and exacerbation of various carcinomas [15, 16]. Thus, we aimed to investigate the promotive effects of Der p 2 on motility and invasiveness of NSCLC cells with emphasis on integrin/FAK-associated signaling cascades.

## RESULTS

### DP2 promotes cell migration and invasion of human NSCLC cells

**Figure 1:**
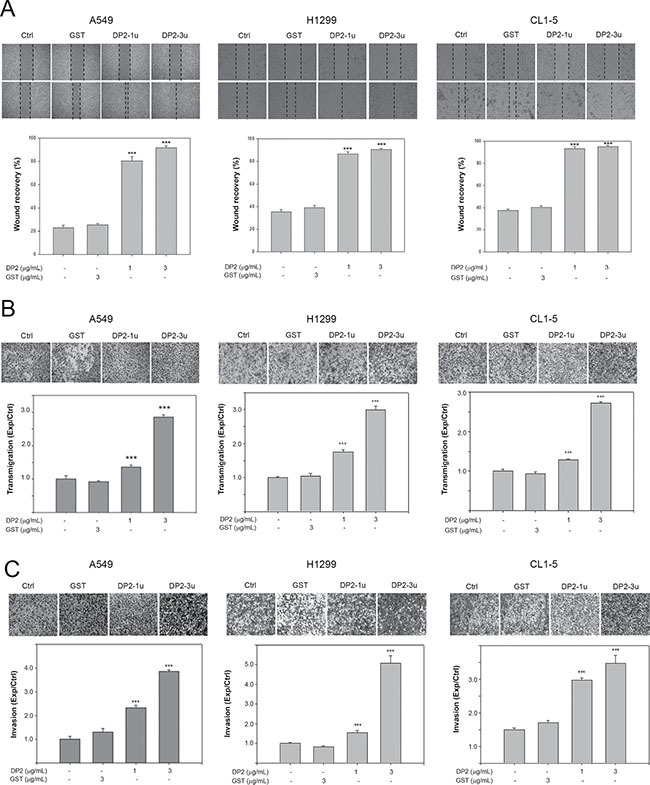
DP2 promotes cell migration and invasion of NSCLC cells (**A**) Wound healing assay was performed in the three cell lines with 48 h of recovery. Cells were cultured on 6-well plates, scratched using tips, and then incubated with GST (3 μg/mL), DP2-1u (1 μg/mL), or DP2-3u (3 μg/mL) for 48 h. After the incubation, wound recovery was determined as comparing to the initial of each treatment group. (**B**) and (**C**), cells were subjected to transmigration and invasion assay with incubation of GST (3 μg/mL), DP2-1u (1 μg/mL), or DP2-3u (3 μg/mL) for 24 h. The transmigrated cells on the bottom side of membrane were stained and counted using light microscope at a magnitude of 200X. Transmigration and invasion were presented as the ratio of treatment/control. Ctrl, control; ***P < 0.005 as compared to GST treatment.

To evaluate whether DP2 could promote lung cancer metastatic ability, we examined the effects of DP2 on cell migration and invasion of 3 different NSCLC cell lines. We first performed would healing assay with A549, H1299, and CL1-5 cell lines. Cells were treated either with glutathione S-transferase (GST), 1 μg/mL DP2, or 3 μg/mL DP2 during the 48 hours (h) of wound-healing period. In all three different cell lines, GST-treated cells showed similar rate of wound-healing as the control cells. DP2-treated cells showed significant increased rate of wound recovery (Figure 1A). The growth rate of these three cancer cell lines were not affected by incubation with up to 5 μg/mL of DP2 (Supplementary Figure 1). We also evaluated the effect of DP2 on cell migration using transmigration assays. As shown in Figure 1B, the transmigration rate of all three cell lines were increased upon incubation with DP2 in a dose-dependent manner. Incubation with 1 μg/mL DP2 resulted in 1.36–1.77 fold increase of the transmigration rate as compared to cells incubated with GST (*P* < 0.005 for the 3 cell lines); incubation with 3 μg/mL DP2 resulted in 2.55–2.86 fold increase of the transmigration rate as compared to GST-treated group (*P* < 0.005 for the 3 cell lines). (Figure 1B). We next tested the invasiveness of these cell lines by evaluating the transmigration of cells through Matrigel. DP2 treated cells showed increased invasiveness through the Matrigel compared to the GST-treated cells. Cells treated with 3 μg/mL DP2 showed 3.5–5.1 fold increase in Matrigel invasion. (Figure 1C). These findings indicated that DP2 significantly promoted cell motility and invasiveness of these NSCLC cells.

### DP2 enhances cell migration and invasion associating with FAK and MAPK pathway

**Figure 2:**
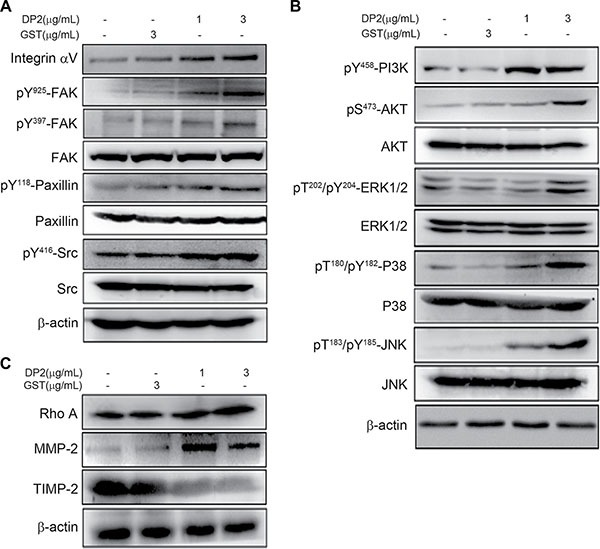
DP2 promoted migration and invasion of A549 cell associating with FAK and MAPK pathway Cells were incubated with GST or DP2 at the indicated concentration for 1 h, and then lysed for immunodetection of determining the level of indicated phosphorylated proteins or total proteins. β-actin was sued as internal control.

Carcinoma invasiveness is highly associated with activation of integrin and its downstream signaling pathway, including FAK, paxillin, Rho and metalloproteinases (MMPs) [17]. We therefore investigated whether DP2 could enhance integrin signaling cascade. We examined the expression and phosphorylation of components in the integrin signaling pathway in A549 cells after DP2 treatment by immunoblotting. As shown in Figure 2A, DP2 treatment increased expression level of integrin αV and triggered phosphorylation of FAK (Y937/Y925), paxillin (Y118) and Src. Src phosphorylation is known to activate phosphatidylinositol 3-kinase (PI3K)/AKT and mitogen-activated protein kinases (MAPKs) such as extracellular signal-regulated kinase (ERK1/2) p38 MAPK (P38) and c-Jun N-terminal kinase (JNK) [18]. We observed that PI3K/AKT and the MAPKs such as ERK1/2, P38 and JNK were activated in response to DP2 treatment (Figure 2B). In addition, Rho A, the downstream signal component of PI3K/AKT and MAPKs that involved in cell invasiveness, and MMP-2 expression were upregulated by DP2 treatment. Meanwhile, expression of tissue inhibitor of metalloproteinase-2 (TIMP-2), an important inhibitor of MMP-2, was downregulated in response to DP2 treatment. Taken together, these results indicated that DP2 treatment of A549 cells upregulated integrin αV expression and triggered FAK/Src signaling. This in turn might have contributed to PI3K/AKT and MAPKs activation, Rho upregulation, and subsequently increased MMP-2 while lowering TIMP-2 expression.

### Involvement of FAK activity in DP2-induced cell invasion

**Figure 3:**
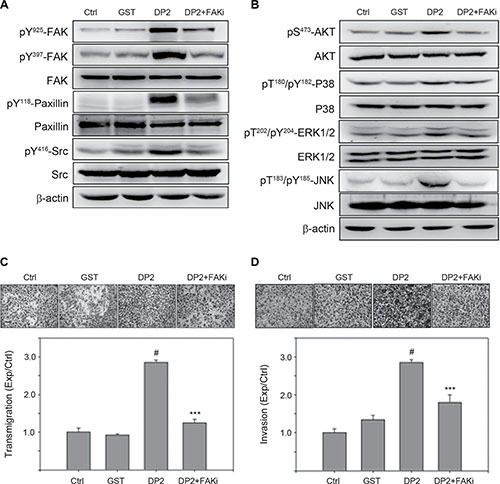
DP2 enhanced migration and invasion of A549 cells through FAK activation (**A**) and (**B**), cells were incubated with GST and DP2 at 3 μg/mL for 1 h or pretreated with FAK inhibitor (FAKi) for 2 h and then treated with DP2 at 3 μg/mL for 1 h. After the treatments, cells were lysed for immunodetection of determining the level of indicated phosphorylated proteins or total proteins. β-actin was sued as internal control. (**C**) and (**D**), cells were seeded on blank or Matrigel-coated transwell inserts, and then incubated with GST and DP2 at 3 μg/mL for 24 h or pretreated with FAK inhibitor (FAKi) for 2 h following treated with DP2 at 3 μg/mL for 24 h. The transmigrated cells on the bottom side of membrane were stained and counted using light microscope at a magnitude of 200X. Transmigration and invasion were presented as the ratio of treatment/control. Ctrl, control. ^#^*P* < 0.05 as compared to GST treatment. ***P < 0.005 as compared to DP2 alone.

Our observation showed that DP2 treatment was able to trigger FAK/Src signaling and activation of PI3K/AKT and MAPKs. We therefore explored the role of FAK signaling in DP2-promoted invasiveness of lung carcinoma cell A549 by using a specific FAK inhibitor 1,2,4,5-Benzenetetraamine. Cells were pretreated with FAK inhibitor for 1 h prior to DP2 incubation and subjected to immunoblotting analysis 30 min later. As shown in Figure 3A, DP2-induced phosphorylation of FAK (Y925/Y397), paxillin (Y118) and Src (Y416) in A549 cells was lowered in FAK inhibitor-treated group as compared to the GST-treated group. In addition, DP2-induced activation of AKT and MAPK including ERK1/2, P38, and JNK in A549 cells was not present in the FAK inhibitor-treated group (Figure 3B). Moreover, DP2-promoted transmigration and invasion ability through Matrigel were significantly diminished in the presence of the FAK inhibitor when compared to DP2 alone, respectively (*P* < 0.005, Figure 3C and 3D). Collectively, these results indicated that the DP2-promoted invasiveness of A549 cell was dependent on the activation of FAK.

### DP2 elevates expression of urokinase-type plasminogen activator (uPA) and uPA receptor (uPAR) and enhances uPAR binding to integrin

**Figure 4:**
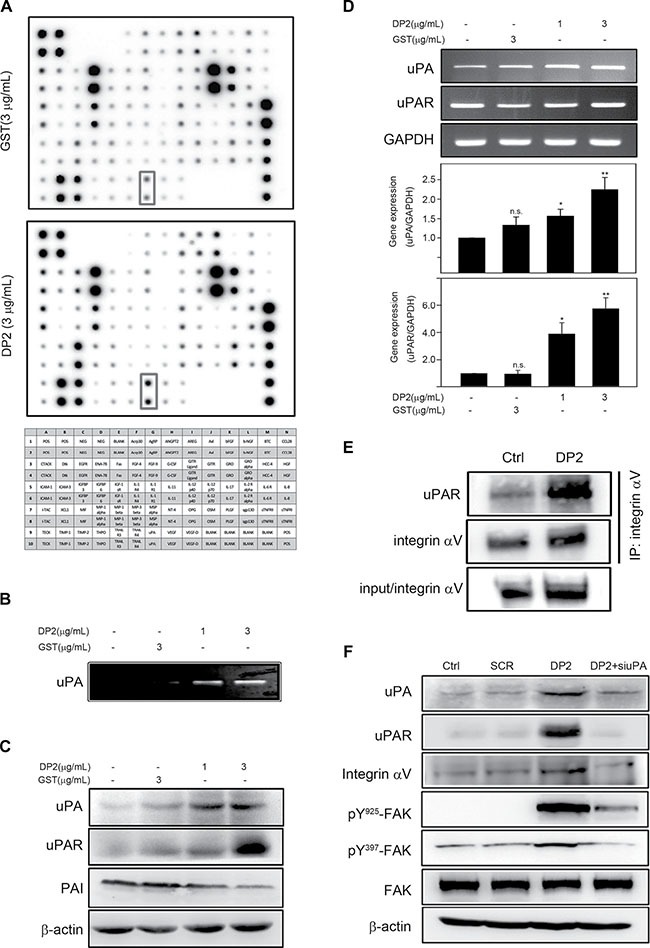
DP2 upregulated uPA/uPAR expression, enhanced uPAR/integrin αV interaction and induced FAK activation (**A**) and (**B**), cells were incubated with GST or DP2 at 3 μg/mL for 24 h, and then the cultured medium was subjected to cytokine array assay or uPA activity assay. (**C**) and (**D**), cells were incubated with GST or DP2 at 3 μg/mL for 1 or 4 h, and then lysed for immunodetection of the indicated proteins or for total RNA extraction and the following mRNA expression analysis using RT-PCR and qRT-PCR. (**E**) cells were incubated with GST or DP2 at 3 μg/mL for 24 h, and then lysed for immunoprecipitation using antibodies against integrin αV. The precipitated proteins were subjected to immunodetection of uPAR and integrin αV. (**F**) cells were transfected with scramble RNA (SCR) or specific siRNA against uPA (siuPA), treated with DP2 at 3 μg/mL for 24 h, and then lysed for immunodetection of determining the level of indicated phosphorylated proteins or total proteins. β-actin was sued as internal control.

Coordinating cell motility and ECM proteolysis is crucial in cancer metastasis [17]. Using protein array, we found that levels of several proteolytic enzymes secreted by A549 cell were elevated in response to DP2 treatment, including uPA and MMP-2 (Figure 4A). One of the proteolytic enzyme that showed increased expression is uPA. The binding of uPA to its receptor uPAR is known to induce uPAR clustering that subsequently triggers uPAR/integrin interaction and integrin/FAK signaling activation [19]. We therefore examined the role of uPA and uPAR in DP2-induced FAK signaling. We incubated A549 cells with GST or DP2 for 24 h, and then analyzed the uPA enzymatic activity in cultured medium and the expression level of uPA, uPAR, and plasminogen activator inhibitor-1 (PAI-1). As shown in Figure 4B, the enzymatic activity of uPA in culture medium was increased in DP2-treated cells. Consistently, the protein level of uPA and uPAR was increased in DP2-treated cells (Figure 4C). To the opposite, PAI-1, an endogenous fast-acting inhibitor of uPA, was reduced in DP2-treated cells (Figure 4C). At mRNA level, similarly, uPA and uPAR was significantly increased upon DP2 treatment (Figure 4D). We next examined the interaction between uPAR and integrin αV by co-immunoprecipitation in DP2-treated cells. We found that there are increased amount of uPAR protein co-immunoprecipitated with integrin αV in DP2-treated cells than in GST-treated cells (Figure 4E). To further confirm the importance of uPA upregulation in DP2-induced FAK activation, we knocked down the uPA expression by specific siRNA against uPA. As shown in Figure 4F, the addition of uPA specific siRNA in DP2-treated cells inhibited the upregulation of uPA and uPAR expression. These cells also showed lower expression level of integrin αV compared to cells treated with DP2 alone. Phosphorylation of FAK at Y925 and Y397 was visibly lower in siRNA-treated cells as well. These results indicated that DP2 treatment could activate uPA and uPAR protein expression, promote uPAR-integrin αV interaction, leading to FAK activation.

### DP2 upregulates uPA, uPAR, and integrin αV expression and induces FAK activation via TLR2/4 and ERK1/2

**Figure 5:**
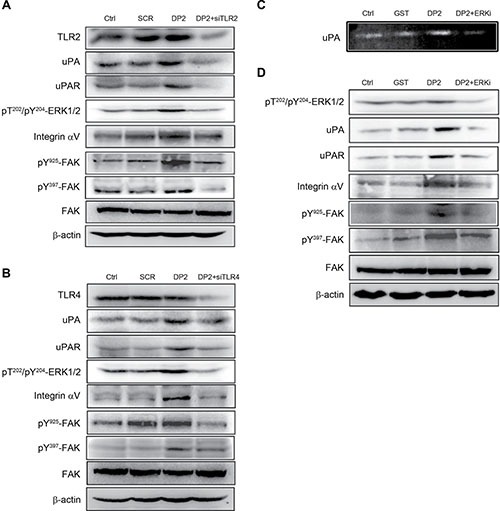
DP2 upregulated uPA/uPAR protein expression and enhanced integrin αV/FAK activation via TLR2/4 and ERK1/2-mediated signaling (**A**) and (**B**) cells were transfected with scramble RNA (SCR) or siRNA against TLR2 and TLR4 (siTLR2 and siTLR4), treated with DP2 at 3 μg/mL for 1 h, and then lysed for immunodetection of determining the level of indicated phosphorylated proteins or total proteins. (**C**) cells were incubated with GST or DP2 at 3 μg/mL for 24 h, or pretreated with ERK inhibitor (ERKi) for 2 h following treated with DP2 at 3 μg/mL for 24 h; then, the cultured medium was collected for uPA activity assay. (**D**) cells were incubated with GST or DP2 at 3 μg/mL for 1 h, or pretreated with ERK inhibitor (ERKi) for 2 h following treated with DP2 at 3 μg/mL for 1 h, and then subjected to immunodetection of determining the level of indicated phosphorylated proteins or total proteins. β-actin was sued as internal control.

Previous studies have demonstrated that TLR2/4 signaling pathway plays a pivotal role in DP2-triggered inflammatory responses [9, 20, 21]. Among the TLR2/4 signaling cascades, activation of ERK1/2 is highly associated with chemokine production. In addition, ERK1/2 activation has been suggested to be involved in regulating uPA and uPAR expression [22]. Therefore, the role of TLR2/4-ERK1/2 signaling cascade in DP2-mediated upregulation of uPA, uPAR, and integrin and FAK activation was further investigated. We used specific siRNA against TLR2 or TLR4 to examine the effect of DP2-treatment on A549 cells in the absence of TLR2/4 signaling. As shown in Figure 5A, DP2 treatment did not alter TLR2 expression level as compared to the control and scramble (SCR) groups. By using specific siRNA against TLR2, TLR2 expression was downregulated. In DP2-treated and TLR2-down regulated cells, ERK1/2 phosphorylation (T202/Y204) was not increased as in the DP2-treated cells. The upregulation of uPA, uPAR, and integrin αV expression as well as the FAK phosphorylation (Y925/Y397) in response to DP2 were also not observed in TLR2 knocked-down cells. The similar inhibitory effects on uPA, uPAR, and integrin expression and FAK phosphorylation in A549 cells were also observed in DP2-treated cells transfected with specific siRNA against TLR4 (Figure 5B). We also examined the role of ERK1/2 activation in cellular responses to DP2-treatment using a specific ERK inhibitor (ERKi, PD98059). As shown in Figure 5C, inhibition of ERK by using specific inhibitor ERKi significantly lowered the DP2-promoted uPA enzymatic activity. In addition, the inhibition of ERK1/2 also interfered with DP2-dependent upregulation of uPA, uPAR, and integrin αV expression and FAK activation (Figure 5D). Taken together, these results showed that the effects of DP2 on uPA/FAK activation is dependent on TLR2/4 and ERK1/2 signaling cascade.

## DISCUSSION

Chronic inflammation, a continued active inflammation response, is known to result in cumulative tissue destruction [23]. Furthermore, chronic inflammation has been generally regarded as a major risk factor that promotes carcinogenesis, tumor growth [24], and tumor metastasis [25] as well as exacerbates various pathophysiological processes, including hypersensitiveness and angiogenesis [26]. Among the chronic inflammation associated mediators, tumor necrosis factor alpha (TNFα), interleukin-6 (IL-6), vascular epidermal growth factor (VEGF), and MMPs are of particular attention for their role in tumor development [27]. Our previous studies have shown that DP2 elevates production of several proinflammatory mediators by human airway epithelial cell including IL-6, IL-8, and monocyte chemoattractant protein-1 (MCP-1) [28].

Inhaled HDM allergens are known to interact with airway epithelial cell and lead to epithelium damage and contribute to airway remodeling [29]. In this study, we demonstrate that DP2 can upregulate uPA and uPAR expression via TLR2/4-mediated ERK activation, reinforce uPAR binding to integrin, and subsequently activate integrin/FAK signal transduction, resulting in the promoted cell motility and invasive ability of NSCLC cell A549. In addition, we also observe that DP2 increases MMP-2 expression while decreasing TIMP-2 and PAI expression. Collectively, these findings suggest that DP2 could promote metastasis of NSCLC.

Integrin is a major class of adhesion receptors involved in regulation of cell migration [30], cell survival [31], and cell growth [32] after binding to their ligands. FAK, integrin-linked kinase (ILK) and Src kinases are the well-studied downstream effectors that were activated in response to integrin activation [33]. The pY397-FAK is recognized by Src-homology 2 (SH2) domain-containing proteins such as Src family kinases (SFK). The pY925-FAK enables binding of growth factor receptor-bound protein 2 (GRB2), and following activates Ras/MAPK pathway [34]. In parallel, SH2 domain of PI3K regulatory subunit p85 binding to pY397-FAK leads to activation of PI3K/Akt signaling cascade [35]. In this study, we demonstrate that DP2-triggered activation of integrins αV via binding to uPAR and the downstream FAK/Src and PI3K/AKT involve in the promoted cell mobility and invasiveness of A549 cell.

In addition to induction of integrin downstream signaling cascade, we observed that DP2 could also upregulate expression level of integrin αV, αVβ5, and αVβ3 (Figure 2A, and Supplementary Figure 2). Several integrin isoforms such αVβ5 and αVβ3 are weakly expressed in normal epithelial tissues, whereas they are highly expressed during epithelial-to-mesenchymal transition (EMT) [36]. Furthermore, the expression of integrin αVβ3 and β3 in human lung fibroblasts is found upregulated in response to transforming growth factor (TGF)-β1 stimuli through Src- and p38 MAPK-dependent pathway [37]. Our previous studies have demonstrated the critical role of DP2-induced AKT/glycogen synthase kinase (GSK-3β)/β-catenin axis and ERK1/2 in increased expression of claudin-2 and EMT inducer Snail and Slug [13, 14]. In this study, we also observe that DP2 can activate FAK/Src, ERK1/2, p38 MAPK, and JNK. Taken together, we suggest that the DP2-upregulated expression of integrin isoforms may attribute to activation of Src/p38 MAPK signaling and induction of EMT.

Recently, mucin 5AC (MUC5AC), a mucin protein that is overexpressed in metastatic lung cancer and over-produced in hypersensitive airway exposed to HDM, is reported interplaying with integrin β4 and subsequently trigger FAK signaling, resulting in enhanced migration of A549 and H1437 [38, 39]. Thus, we suggest that Der p 2 may also promote motility of lung carcinoma cells via upregulation of MUC5AC. However, further investigation is needed. Collectively, these findings imply that inhalation of DP2 not only reinforces invasiveness of lung carcinoma cell via activation of integrin/FAK signaling but also may promote lung carcinoma metastasis via increasing production of tumor development mediators such as uPA, MMP-2, and IL-6.

MAPK pathway plays an important role in the regulation of MMP expression and metastasis of tumor cells [40]. TIMP-2 is an inhibitor against metalloproteinases via direct binding [41]. A recent study reports that ERK1/2 and AKT activation induced by G protein-coupled receptor and epidermal growth factor receptor is highly associated with elevated expression of MMP-2 and MMP-9 and reduced expression of TIMP-2 and E-cadherin expression in gastric carcinoma, and the association has clinicopathological significance [42]. Similarly, our results showed that DP2 induced ERK and AKT activation and increased MMP-2 expression and enzymatic activity (Supplementary Figure 3) while decreasing TIMP-2 expression. These observations implicate that DP2 might promote lung carcinoma progression via upregulating the invasive and metastatic mediators. However, further investigation is needed to evaluate the *in vivo* effects of DP2 on lung carcinoma metastasis.

TLR2/4 signaling has been reported involving in tumor cell immune escape and tumor progression in inflammatory microenvironment [43]. Recent studies have revealed that TLRs are highly expressed by many human tumors, including lung cancer [44], prostate cancer [45], breast cancer [46] and hepatocellular carcinoma [47]. Among the TLRs, TLR2 signaling can promote lung cancer cell growth and lead to reinforced invasiveness and metastasis, and TLR4 signaling can mediate metastasis via enhancing tumor cell invasion, proliferation, and survival of prostate cancer cells. DP2 is known to trigger innate immunity through direct interaction with and activation of TLR2 and TLR4, which subsequently contributes to activation of ERK pathway. In metastatic carcinoma cells, ERK1/2 activity was highly elevated as compared to non-metastatic carcinoma cells [48]. In addition, ERK signaling plays an important role in uPAR/integrin-mediated upregulation of colon cancer cell motility [49]. Overexpression of uPAR has been demonstrated to be essential for tumor invasion and metastasis in different carcinoma cells [50, 51]. Here, our results show that DP2 can upregulate uPA/uPAR expression, which may attribute to TRL2/4-mediated ERK signaling. These findings indicate that DP2-induced TLR2/4 activation promotes uPA/uPAR expression and the following activation of integrin/FAK signaling cascade, subsequently contributing to the enhanced cell motility and invasiveness of lung carcinoma cell.

**Figure 6:**
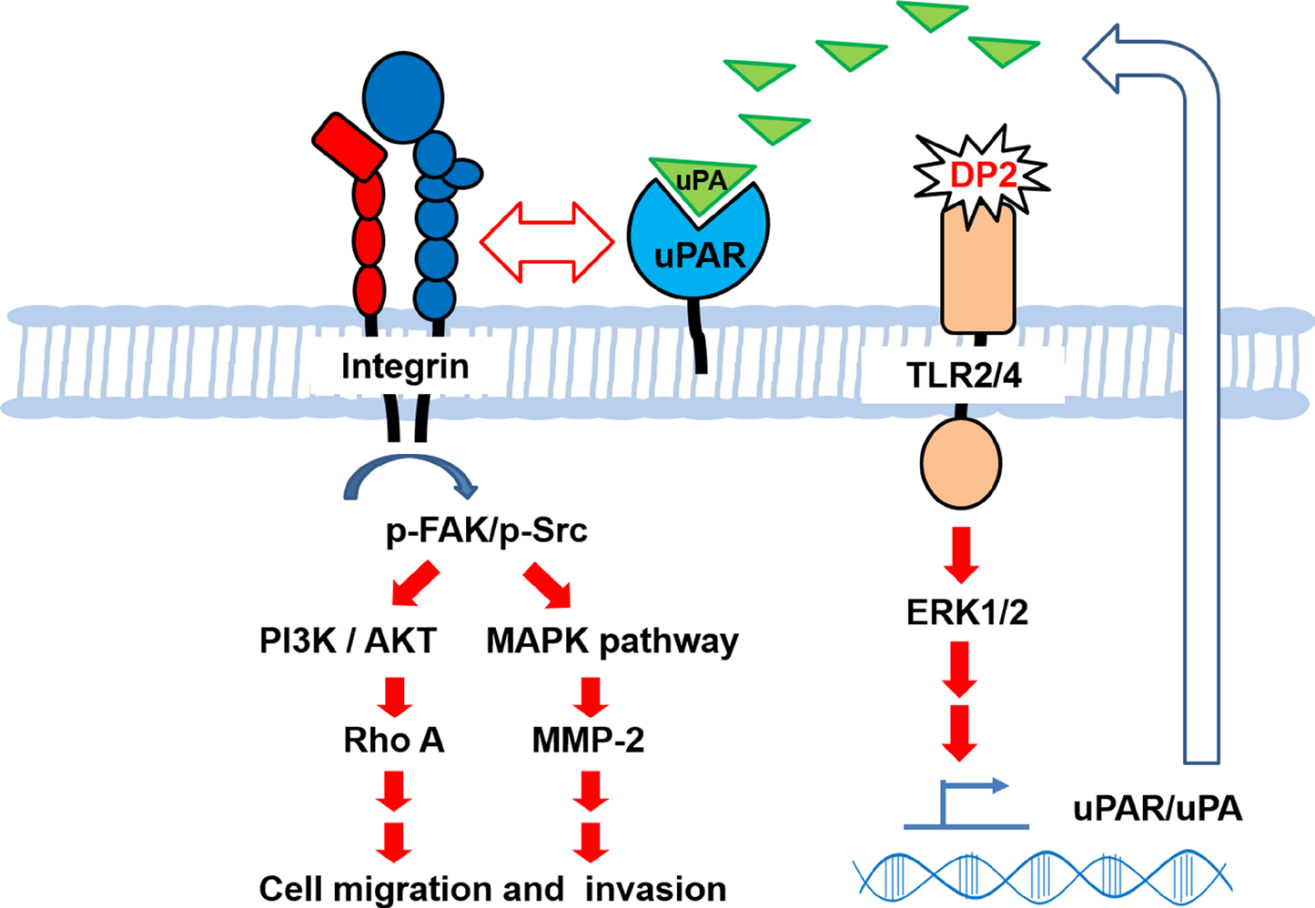
Proposed mechanism for upregulated cell motility and invasiveness of A549 cell induced by DP2 Our findings demonstrate that DP2 induces TLR2/4-ERK1/2 signal cascade that upregulates uPAR/uPA expression and secretion, contributing to enhancement of uPAR/integrin αV interaction, triggering the downstream FAK/Src, PI3K/AKT, and MAPK activation, increasing Rho A and MMP-2 expression, and consequently promoting cell migratory and invasive ability of NSCLC cell A549.

In conclusion, we demonstrate that DP2 promotes cell motility and invasiveness of human lung carcinoma cells, which attributes to the upregulated expression of uPA/uPAR and MMP-2 and activation of FAK/integrin signaling axis via TLR2/4-mediated ERK activation (Figure 6). Accordingly, we propose that the indoor aeroallergen DP2 may be regarded as a potential risk factor for lung cancer, which might aggravate lung carcinoma via enhancing its metastatic ability.

## MATERIALS AND METHODS

### Expression and purification of recombinant DP2

DP2 was a recombinant fusion polypeptide with an N-terminal glutathione S-transferase (GST) tag. Expression of DP2 and GST protein was achieved by using Escherichia coli BL-21 (Novagen, Madison, WI, USA) strain containing pGES-GST-DP2 plasmid and pGES-GST (a gift from Dr. Jiunn-Liang Ko), respectively. *E. coli* was cultured at 37°C and protein expression was induced by using 0.1 mM isopropyl β-D-thiogalactoside. The expressed DP2 fusion protein and GST control protein was purified by glutathione affinity chromatography and following by Superdex 75 column (Amersham-Pharmacia Biotech AB, Upsala, Sweden). The purified protein concentration was determined by Bradford protein assay kit. Endotoxin in purified protein was determined by using Pierce™ LAL Chromogenic Endotoxin Quantitation Kit (Thermo Fisher Scientific, Rockford, IL, USA).

### Cell culture and treatments

Human NSCLC adenocarcinoma cell line A549, H1299, and CL1-5 were obtained from ATCC (Manassas, VA, USA) and Prof. Pan-Chyr Yang of National Taiwan University [52], respectively. Cells were cultured in Dulbecco's modified Eagle's medium supplemented with 10% fetal bovine serum (FBS, Invitrogen, Carlsbad, CA, USA), 2 mM glutamine, 100 U/mL penicillin and 100 mg/mL streptomycin (Sigma-Aldrich, St. Louis, MO, USA), and maintained at 37°C in a humidified atmosphere of 5% CO_2_. Prior to DP2 treatments, attachment of cells was achieved by incubating cells with DMEM containing 1% serum for 16 hours (h). DP2 treatments were performed by incubated the attached cells with serial concentrations of DP2 for 1 h (for phosphorylation level), 4 h (for mRNA expression) or for 24 h (for protein expression). Inhibition of FAK or ERK was performed by pretreating cells with individual specific inhibitor (FAKi, 1,2,4,5-Benzenetetraamine. 4HCl; ERKi, PD98059; Calbiochem, La Jolla, CA, USA) for 1 h prior to the DP2 treatments. Cells incubated with the purified GST protein were used as mock control.

### Wound healing migration assay

Cells were seeded in 6-well plates at an initial density of 1 × 10^6^ cells/mL and cultured until 80% confluency. After fasting cells with serum-free medium for 16 h, a line in middle area of well was scratched using a sterile 10 μL-pipette tip. Cells migrated into the wound area was photographed at 0 h and 48 h using a microscope and the acquired images were analyzed by qCMA software for determination of wound recovery [53].

### Cell transmigration and invasion assays

After starving with serum-medium for 16 h, the surviving cells were harvested, resuspended in serum-free medium containing DP2 at the indicated concentrations, and then seeded to the upper chamber (1 × 10^5^ cells/mL) and meanwhile, medium containing 20% FBS was added to the bottom chamber (Millicell invasion chambers, 8 mm pore size, Millipore, Bedford, MA, USA). The chamber was transferred into an incubator with air supplement containing 5% CO_2_ and constant temperature at 37°C, and then incubated for 24 h. After the incubation, the tumor cells in the bottom side of membrane were stained with 1% crystal violet solution and counted using a microscope (200×).

### Casein zymography and cytokine array

After a treatment with DP2 for 24 h, conditioned media (CM) were collected, and then subjected to 0.2% casein-8% SDS-PAGE to determine the uPA activity. Gels were incubated in 2.5% Triton X-100 at room temperature for 2 h and subsequently at 37°C overnight in the activating buffer (50 mM Tris, pH 7.5, 10 mM CaCl_2_, 0.15 M NaCl). After the incubation, gels were stained with Coomassie Blue R-250 for detection of proteinase activity.

RayBio^TM^ Human Cytokine Array (Raybiotech, Norcross, GA, USA) was used for detection of cytokines that were differentially expressed in control-CM comparing to that of DP2 treated-CM. One hundred micrograms of CM was used for analysis according to manufacturer's instructions. Signals were developed by using ECL Chemiluminescent kit and analyzed using Multi Gauge software.

### Immunoprecipitation and Western blot

Cells were lysed in lysis buffer containing protease inhibitors, and then the cell lysates were centrifuged at 20,000 g for 15 min to remove the debris. The clarified supernatants were immunoprecipitated with antibody to integrin αV and the immunoprecipitates were blotted for uPAR and integrin αV. After extraction, cell lysates were adjusted to an equal amount of protein (50 μg), electrophoresed in a 10% SDS-PAGE, and transferred onto a nitrocellulose membrane. After blocking with 5% nonfat milk/PBS containing 0.5% Tween-20, the membranes were incubated with the primary antibodies at a 1:1000 dilution at 4°C for 16 h. The reacted membrane was washed using PBS containing 1% Tween-20, and then incubated with goat anti-rabbit or anti-mouse IgG conjugated with horseradish peroxidase for 1 h. Signals were developed by using ECL Chemiluminescent kit and analyzed using Multi Gauge software.

### RNA extraction

Total RNA was isolated from lung cancer cells by using RNeasy mini kit (QIAGEN, CA, USA) and DNase I treatment according to the manufacturer's instructions. First strand cDNA was synthesized by reverse transcription in a 20 mL reaction using SuperScript III RTS first-strand cDNA synthesis kit in accordance with the manufacturer's directions. All PCR assays were performed in a 50 μL reaction mixture containing 2.5 μg cDNA and 200 nM of each primer using PCR master mix. The temperature cycle profile for the PCR reactions was 94°C for 5 min, 35 cycles of 94°C for 30 s, 60°C for 30 s, and 72°C for 30 s followed by 72°C for 7 min. The RT-PCR was performed by using the following primers: human uPA, 5′-CAC GCA AGG GGAG ATG AA-3′ (forward) and 5′-ACA GCA TTT TGG TGG TGA CTT-3′ (reverse); human uPAR, 5′-TTA CCT CGA ATG CAT TTC CT-3′ (forward) and 5′-TAT GGT AAG AGG CTG TGC AA-3′ (reverse); glyceraldehyde-3-phosphate dehydrogenase (GAPDH), 5′-CAA GGT CAT CCA TGA CAA CTT TG-3′ (forward) and 5′-GTC CAC CAC CCT GTT GCT GTA G-3′ (reverse). The PCR products were analyzed by using 2% agarose electrophoresis.

### Quantitative real-time reverse transcription-PCR (qRT-PCR)

qRT-PCR was performed for gene expression quantitation by using the ABI PRISM 7700 sequence detection system (Applied Biosystems, Foster City, CA, USA). FastStart Universal SYBR Green Master (Roche Applied Science, Mannheim, Germany) was used for Taqman PCR. Relative gene expressions were calculated by using the 2^–ΔΔCt^ method [54]. The qRT-PCR was performed by using the following primers: human uPA, 5′-CCC AGA TCG AGA CTC AAA GC-3′ (forward) and 5′-GAC CCA TTC CCA AAG TAG CA-3′ (reverse); human uPAR, 5′-GCC CAA TCC TGG AGC TTG A-3′ (forward) and 5′-TCC CCT TGC AGC TGT AAC ACT-3′ (reverse); GAPDH, 5′-CAA TGA CCC CTT CAT TGA CC-3′ (forward) and 5′-GAC AAG CTT CCC GTT CTC AG-3′ (reverse). The Gapdh gene was used an endogenous internal control. Triplicate qRT-PCR experiments were performed for each sample. The size of the PCR products was confirmed as correct by agarose gel electrophoresis.

### RNA interference

Cells were seeded on 6 cm-dishes and transfected with a final concentration of 30 nm specific small interfering RNA (siRNA) oligonucleotides against TLR2 or TLR4 (Genedirex, NV, USA) by using Lipofectamine RNAiMAX Reagent (Invitrogen, Carlsbad, CA, USA) according to the manufacturer's protocol. A non-targeting siRNA was used as control. Control non-silencing siRNA (sense 5′-UUC UCC GAA CGU GUC ACG UTT-3′, antisense 5′-ACG UGA CAC GUU CGG AGA ATT-3′) TLR2 siRNA targeting (sense 5′-GCC UUG ACC UGU CCA ACA ATT-3′, antisense 5′-UUG UUG GAC AGG UCA AGG CTT-3′) and TLR4 siRNA targeting (sense 5′-CCA GGU GCA UUU AAA GAA ATT-3′, antisense 5′-UUU CUU UAA AUG CAC CUG GTT-3′) were purchased from Genedirex (Nevada, USA).

### Statistical analysis

Data were expressed as mean SEM of the three independent experiments. Statistical significance analysis was determined by using one-way ANOVA followed by Dunnett for multiple comparisons with the control or the impaired two-tailed Student's *t*-test. *P* < 0.05 was considered as significant.

## SUPPLEMENTARY MATERIALS FIGURES AND TABLES


