# Supplementary material for: Aeroallergen Der p 2 promotes motility of human non-small cell lung cancer cells via toll-like receptor-mediated up-regulation of urokinase-type plasminogen activator and integrin/focal adhesion kinase signaling
Source: Oncotarget. 2017 Jan 5;8(7):11316–28. doi: 10.18632/oncotarget.14514 (PMC5355267; doi:10.18632/oncotarget.14514)
Supplement: Supplementary file 1 [file oncotarget-08-11316-s001.pdf]

Aeroallergen Der p 2 promotes motility of human non-small cell lung cancer cells via toll-like receptor-mediated up-regulation of urokinase-type plasminogen activator and integrin/focal adhesion kinase signaling

Supplementary Materials

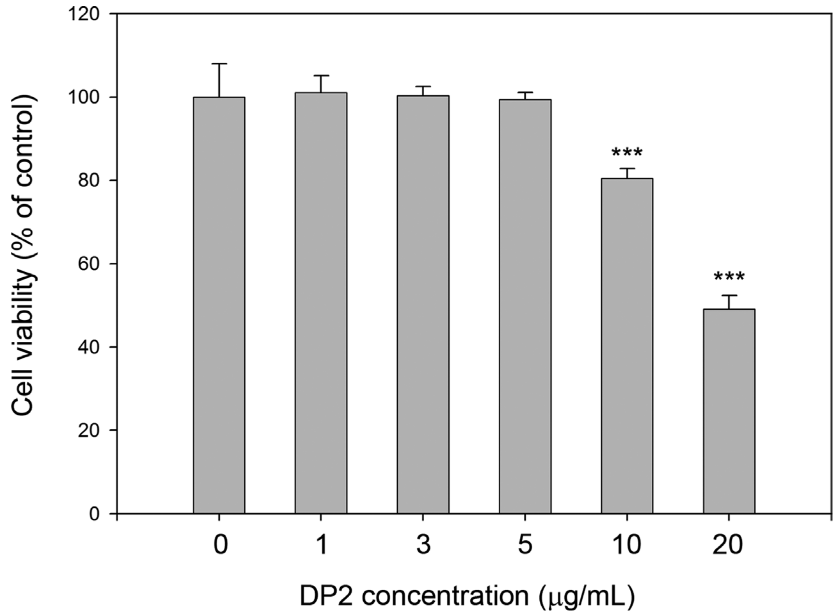

Supplementary Figure 1: Effects of DP2 on cell viability of NSCLC A549 cell. Cells at the density of 10^5 per mL were treated with a serial concentrations of DP2 as indicated for 24 hours. After the treaments, cell viability was assessed by using MTT assay and presented as persentage of contorl. ****P* < 0.005 as compared to control.

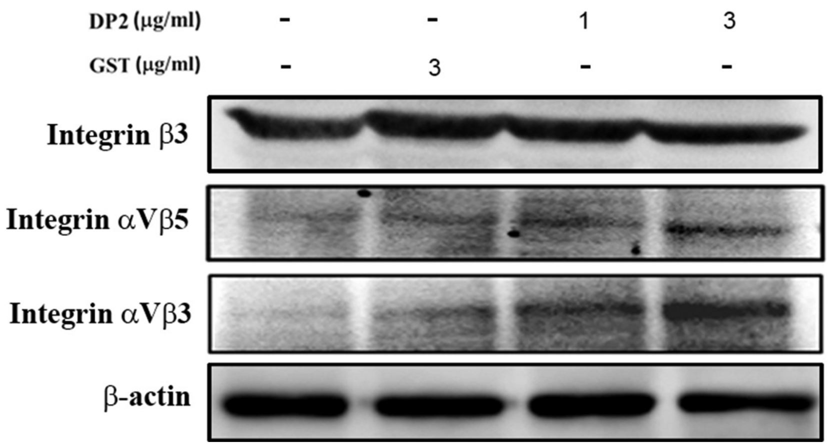

Supplementary Figure 2: Effects of DP2 on expression level of intergin isoforms in NSCLC A549 cell. Cells at the density of 5×10^5 per mL were treated with 3 mg/mL GST, or 1 or 3 mg/mL DP2 for 24 hours. After the treatments, the cells were collected and lysed for immunodetection of integrin isoforms (β3, αVβ5, αVβ3) by using specific antibodies. Signal of β-actin was used as internal control.

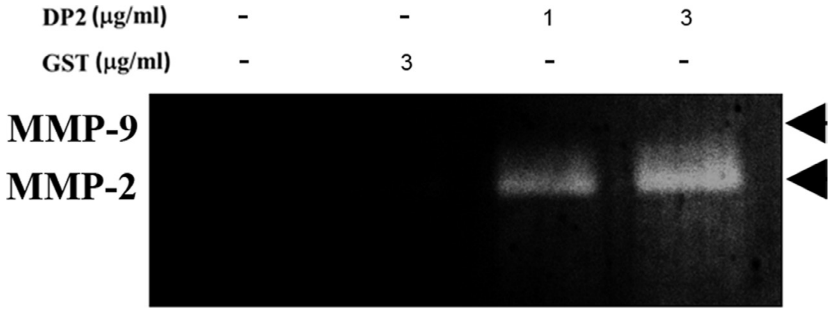

Supplementary Figure 3: Effects of DP2 on enzymatic activity of MMP-2 and MMP-9 secreted by NSCLC A549 cell. Cells at the density of 5×10^5 per mL were treated with 3 µg/mL GST, or 1 or 3 µg/mL DP2 for 24 hours. After the treatments, the cultured medium were collected for MMP proteolitic activity by using zymography. MMP-2 and MMP-9 were indicated.
